# Partially Renewable Poly(butylene 2,5-furandicarboxylate-*co*-isophthalate) Copolyesters Obtained by ROP

**DOI:** 10.3390/polym10050483

**Published:** 2018-04-28

**Authors:** Juan Carlos Morales-Huerta, Antxon Martínez de Ilarduya, Sebastián Muñoz-Guerra

**Affiliations:** Department d’Enginyeria Química, Universitat Politècnica de Catalunya, ETSEIB, Diagonal 647, 08028 Barcelona, Spain; juan.carlos.morales.huerta@upc.edu

**Keywords:** PBF, PBI, copolyesters, ROP, cyclic oligomers, thermal properties, crystallization

## Abstract

Cyclic butylene furandicarboxylate (*c*(BF)_n_) and butylene isophthalate (*c*(BI)_n_) oligomers obtained by high dilution condensation reaction were polymerized in bulk at 200 °C with Sn(Oct)_2_ catalyst via ring opening polymerization to give homopolyesters and copolyesters (*co*PBF_x_I_y_) with weight average molar masses in the 60,000–70,000 g·mol^−1^ range and dispersities between 1.3 and 1.9. The composition of the copolyesters as determined by NMR was practically the same as that of the feed, and they all showed an almost random microstructure. The copolyesters were thermally stable up to 300 °C and crystalline for all compositions, and have *T*_g_ in the 40–20 °C range with values decreasing almost linearly with their content in isophthalate units in the copolyester. Both melting temperature and enthalpy of the copolyesters decreased as the content in butylene isophthalate units increased up to a composition 30/70 (BF/BI), at which the triclinic crystal phase made exclusively of butylene furanoate units changed to the crystal structure of PBI. The partial replacement of furanoate by isophthalate units decreased substantially the crystallizability of PBF.

## 1. Introduction

Due to popular awareness of sustainability, polymers obtained from renewable sources have been developed in the last decade, with the purpose of replacing those obtained from fossil resources [1,2,3,4,5]. 

One renewable monomer that has attracted much attention is 2,5-furandicarboxylic acid (FDCA), an aromatic building block obtained from C5 and C6 sugars, that is able to replace terephthalic acid, a petrochemical compound widely used for the preparation of aromatic polyesters such as PET or PBT [6,7,8]. Poly(ethylene furanoate) (PEF) has been extensively studied because it has not only similar properties to PET, but also improved gas barrier properties, which make it a serious alternative for applications in soft drink bottles. In contrast, poly(butylene furanoate) (PBF) has been much less studied; as such, the knowledge available on this polyester is relatively scarce. PBF is a semicrystalline polymer with a melting temperature of 172 °C and a glass transition temperature of 39 °C [9]. As with PBT, the presence of the butylene segment in the repeating unit of PBF confers to this polymer a strong propensity for rapid crystallization, which is inconvenient for some injection molding processes due to the excessive mold shrinkage. In order to overcome these problems, one solution is copolymerization. The insertion of either a diol or diacid comonomeric unit in small quantities in the PBF chain decreases both the melting temperature and enthalpy, therefore reducing processing costs. Copolymerization has been applied to various technical polyesters in order to tune their thermal properties, such as crystallizability, melting or glass transition temperatures [10]. PBF copolyesters with enhanced biodegradability have already been prepared by either melt polycondensation [11,12,13,14] or ring opening polymerization (ROP) of cyclic oligomers [15,16]. This last method, which uses cyclic oligomers for the synthesis, has some advantages because it does not require by-product removal during reaction, implies small or no heat exchange, and attains very high-molecular-weight polymers in reaction times of minutes. ROP for these systems was first examined in detail by Brunelle [17] and recently reviewed by Hodge [18] and Strandman et al. [19]. The technique has been successfully used by us to prepare various PEF and PBF copolyesters with enhanced properties [20,21].

**Scheme 1:**
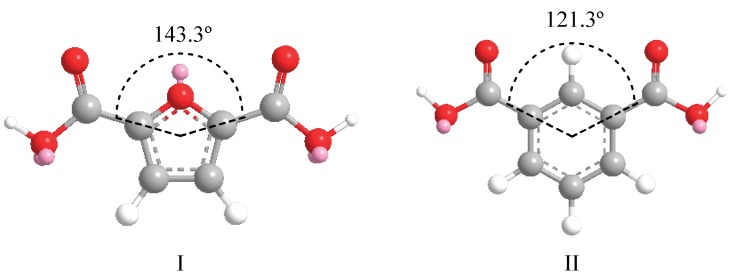
3D models of 2,5-furandicarboxylic acid (I) and isophthalic acid molecules (II).

In this work we would like to report on the synthesis and characterization, evaluation of thermal properties, and crystallization behavior of new, partially renewable PBF copolyesters containing isophthalate units that are prepared by ROP of mixtures of cyclic butylene furanoate and butylene isophthalate oligomers. The 3D chemical structures of FDCA and isophthalic acid (IPA) are depicted in Scheme 1.

## 2. Materials and Methods

### 2.1. Materials

2,5-Furandicarboxylic acid (FDCA, >98% purity) was purchased from Satachem (Shanghai, China). Isophthalic acid (IPA, 99%), 1,4-butanediol (BD), thionyl chloride (SOCl_2_, 99%), and 1,4-di-azabicyclo[2.2.2]octane (DABCO, 99%) and tin(II) ethylhexanoate (Sn(Oct)_2_, 99%) catalysts were purchased from Sigma-Aldrich Co. Triethylamine (Et_3_N, 98%) was purchased from Panreac. Solvents used for reaction, isolation and purification were of high-purity grade and used as received except tetrahydrofuran (THF) that was dried on 3 Å-molecular sieves. The DABCO catalyst was purified by sublimation.

### 2.2. Methods

^1^H- and ^13^C-NMR spectra were recorded on a Bruker AMX-300 spectrometer (Billerica, MA, USA) at 25 °C, operating at 300.1 and 75.5 MHz, respectively. For NMR analysis, monomers, cyclic oligomers and intermediate compounds were dissolved in deuterated chloroform (CDCl_3_) and polymers in pure CDCl_3_ or in a mixture of trifluoroacetic acid (TFA) and CDCl_3_ (1:8). About 10 and 50 mg of sample in 1 mL of solvent were used for ^1^H- and ^13^C-NMR, respectively. Sixty-four scans were recorded for ^1^H, and between 1000 and 10,000 scans for ^13^C-NMR. Spectra were internally referenced to tetramethylsilane (TMS).

High-performance liquid chromatography (HPLC) analysis was performed at 25 °C in a Waters apparatus equipped with a UV detector of Applied Biosystems operating at 254 nm wavelength, and a Scharlau Science column (Si60, 5 μm; 250 × 4.6 mm). Cyclic oligomers (1 mg) were dissolved in chloroform (1 mL) and eluted with hexane/1,4-dioxane 70/30 (*v*/*v*) at a flow rate of 1.0 mL·min^−1^. Molecular weight analysis was performed by GPC on a Waters equipment provided with RI and UV detectors. 100 μL of 0.1% (*w*/*v*) sample solution were injected and chromatographed with a flow of 0.5 mL·min^−1^ of 1,1,1,3,3,3-hexafluoroisopropanol (HFIP). HR5E and HR2 Waters linear Styragel columns (7.8 mm × 300 mm, pore size 103–104 Å) were packed with crosslinked polystyrene and protected with a precolumn. Molar mass average and distributions were calculated against PMMA standards.

Matrix-assisted laser desorption/ionization time of flight (MALDI-TOF) mass spectra were recorded in a 4700 Proteomics Analyzer instrument (Applied Biosystems, Foster City, CA, USA) at the Proteomics Platform of Barcelona Science Park, University of Barcelona. Spectra acquisition was performed in the MS reflector positive-ion mode. About 0.1 mg of sample was dissolved in 50 μL of DCM and 2 μL of this solution was mixed with an equal volume of DCM solution of anthracene (10 mg·mL^−1^); the mixture was then left to evaporate to dryness onto the stainless steel plate of the analyzer. The residue was then covered with 2 μL of a solution of 2,5-dihydroxibenzoic acid in acetonitrile/H_2_O (1/1) containing 0.1% TFA, and the mixture was left to dry prior to exposition to the laser beam.

The thermal behavior of cyclic compounds and polymers were examined by differential scanning calorimetry (DSC), using a Perkin-Elmer Pyris 1 apparatus (Waltam, MA, USA). The thermograms were recorded from 3 to 6 mg samples at heating and cooling rates of 10 °C·min^−1^ under a nitrogen flow of 20 mL·min^−1^. Indium and zinc were used as standards for temperature and enthalpy calibration. The glass transition temperature (*T*_g_) was taken as the inflection point of the heating DSC traces recorded at 20 °C·min^−1^ from melt-quenched samples, and the melting temperature (*T*_m_) was taken as the maximum of the endothermic peak appearing on heating traces. Thermogravimetric analyses were performed on a Mettler-Toledo TGA/DSC 1 Star System under a nitrogen flow of 20 mL·min^−1^ at a heating rate of 10 °C·min^−1^ and within a temperature range of 30 to 600 °C. X-ray diffraction patterns from powdered samples coming directly from synthesis were recorded on a PANalytical X’Pert PRO MPD θ/θ diffractometer using the CuKα radiation of wavelength 0.1542 nm.

### 2.3. Synthesis

#### 2.3.1. Synthesis of Cyclic Oligomers

Cyclic oligomers of butylene 2,5-furandicarboxylate *c*(BF)_n_ and butylene isophthalate *c*(BI)_n_ were synthesized by high dilution condensation (HDC) from equimolar mixtures of BD and furandicarboxylic dichloride (FDCA-Cl_2_) and isophthaloyl chloride (IPA-Cl_2_), respectively, as previously reported Brunelle et al. [22], and more recently by us [23]. Briefly, a three necked round bottom flask charged with 250 mL of THF was cooled to 0 °C; 12.5 mmol (1.40 g) of DABCO was then added under stirring. 5 mmol (0.96 g) of FDCA-Cl_2_ or 5 mmol (1.01 g) of IPA-Cl_2_ in 10 mL and 5 mmol (0.46 g) of BD in THF were drop-wise added simultaneously for 40 min using two addition funnels, in order to maintain the reagents equimolarity in the reaction mixture. The reaction was finished by adding 1 mL of water, followed by 5 mL of 1M HCl; after stirring for 5 min, the mixture was diluted with DCM and filtered. The filtrate was washed with 0.1M HCl, dried on MgSO_4_, and evaporated to dryness to render a mixture of linear and cyclic oligomers. Linear oligomers were removed by chromatography through a short column of silica gel using a cold mixture of DCM/diethyl ether 90/10 (*v*/*v*) as eluent. *c*(BF)_n_: ^1^H-NMR (*δ* ppm, CDCl_3_, 300 MHz): 7.23, 7.24, 7.25 (3s, 2H), 4.40 (m, 4H), 1.92, 1.99 (2m, 4H), ^13^C-NMR (*δ* ppm, CDCl_3_, 75.5 MHz): 158.1, 157.9, 146.7, 146.5, 118.7, 118.6, 118.5, 65.0, 64.8, 25.4. *c*(BI)_n_: ^1^H-NMR (*δ* ppm, CDCl_3_, 300 MHz): 8.62, 8.60 (2m, 1H), 8.26, 8.21 (2m, 2H), 7.56, 7.50 (2m, 1H), 4.45 (m, 4H), 2.01, 1.97 (2m, 4H). ^13^C-NMR (*δ* ppm, CDCl_3_, 75.5 MHz): 165.6, 165.5, 134.2, 133.8, 130.6, 130.5, 130.3 129.7, 128.8, 128.6, 64.8, 64.7, 64.6, 25.5, 25.4.

#### 2.3.2. Synthesis of Polymers

Mixtures of cyclic oligoesters (*c*(BF)_n_ and *c*(BI)_n_) at different molar ratios were polymerized following the procedure used by us for the synthesis of other PBF copolyesters [15,21]. A total of 47 mmol of the mixture of cyclic oligomers with the selected composition together with 0.5 mol % of Sn(Oct)_2_ were dissolved in 10 mL of CHCl_3_, the solution evaporated, and the remaining solid dried under vacuum at room temperature for 24 h. Subsequently, the mixture was left to react in a three necked round bottom flask for 6 h at 200 °C under a flow of N_2_. For optimization of the reaction, the 50:50 mixture was polymerized at 180, 200 and 230 °C. The evolution in the molecular weight of all reactions was monitored by drawing aliquots at different times and analyzing them by GPC. The resulting polymers—without further treatment—were analyzed by NMR, GPC, TGA, DSC and WAXS.

## 3. Results and Discussion

**Figure 1:**
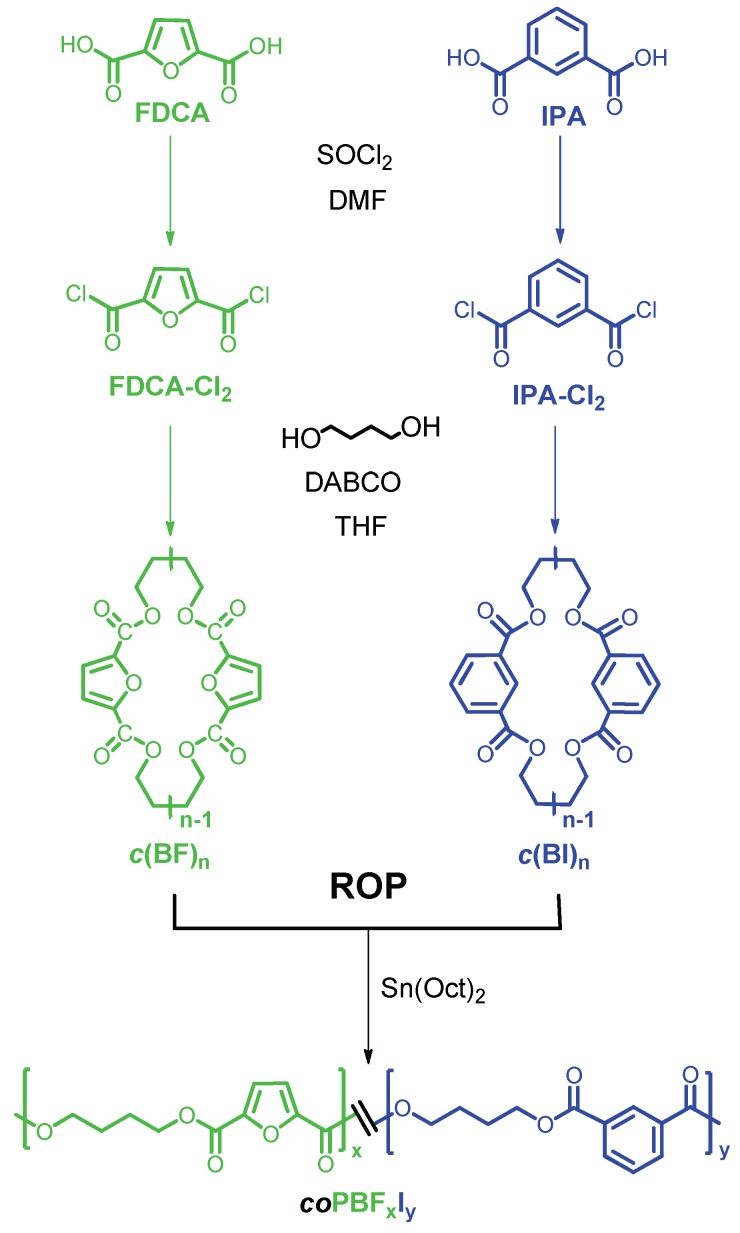
Synthesis route to poly(butylene 2,5-furandicarboxylate-*co*-isophthalate) (*co*PBF_x_I_y_) via ROP.

The synthetic route used for the preparation of *c*(BF)_n_ and *c*(BI)_n_ cyclic oligomers and their ROP is represented in Figure 1. In a first step, the cyclic oligomers were obtained by HDC of BD and either FDCA-Cl_2_ or IPA-Cl_2_. The mixture of cyclic oligomers and linear species were separated by column chromatography, and the purity of cyclic oligomers was ascertained by HPLC, NMR and MALDI-TOF mass spectra (Figures S2 and S3).

The ^1^H-NMR spectra show the absence of any peaks at around 3.8 ppm due to the presence of CH_2_OH groups, which indicates that only cyclic oligomers are present in the purified fractions. Some signals in both ^1^H- and ^13^C-NMR spectra are split due to the sensitivity of these nuclei to the size of the oligomeric cycle. On the other hand, MALDI-TOF MS spectra allowed determining the molar mass of the different cyclic species.

Table 1 shows the composition of the different cycles for the two oligomeric fractions as determined by HPLC. A mixture of cyclic oligomers, mainly from dimer to tetramer, were obtained for both *c*(BF)_n_ and *c*(BI)_n_ being the dimer the predominant cycle size. Both the flexibility of the butylene unit and the 1,3- or 2,5-substitution in benzene or furan respectively, favor the cyclization reaction due to the probable low ring strain of the cycles made of two repeating units.

The thermal properties of these cycles were evaluated by DSC and TGA (Figure S4 and Table 1). Both *c*(BF)_n_ and *c*(BI)_n_ showed melting peaks at around 150 °C, and it was observed that they were thermally stable up to 276 °C and 330 °C, respectively, which allowed their thermal polymerization at the temperature used for reaction (200 °C) without perceiving degradation.

**Figure 2:**
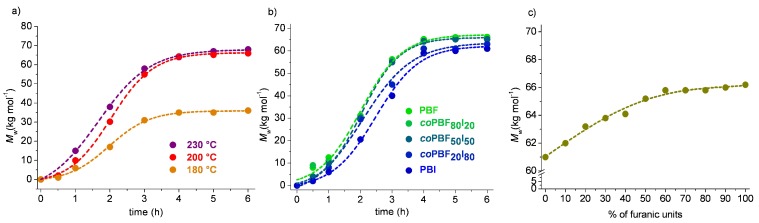
(**a**) Evolution of *M*_w_ of *co*PBF_50_I_50_ with reaction time at different temperatures; (**b**) Evolution of *M*_w_ of *co*PBF_x_I_y_ with reaction time at 200 °C for different compositions; (**c**) Effect of composition of *co*PBF_x_I_y_ on the *M*_w_ of the copolyester produced at 200 °C after 6 h of reaction.

These cycles were then polymerized via ROP in bulk. First, an equimolar mixture of the two cyclic fractions was made to react in order to test the effect of time and temperature on the polymerization results. Three different temperatures above the melting point of the cycles were chosen, i.e., 180, 200 and 230 °C, and sample aliquots were drawn at scheduled periods to determine the evolution of the molar mass of the copolymer produced under different conditions. It was found that the molar mass of the polymer did not increase after six hours of reaction at above 200 °C, (Figure 2a); such a temperature was then chosen for carrying out all copolyesters synthesis. The evolution of the molar mass of the copolyesters with time of reaction is depicted in Figure 2b, where a similar tendency is observed for all the series. However the maximum molar mass attained was observed to increase slightly with the content in furanoate units in the copolyester (Figure 2c and Table 2), which can be due to the higher reactivity of the furanoate over isophthalate cyclic oligomers or the higher thermal stability of the former. The dispersities of the obtained copolyesters oscillated between 1.30 and 1.78 values, which are in accordance with those obtained by entropically driven ROP [18,19].

**Figure 3:**
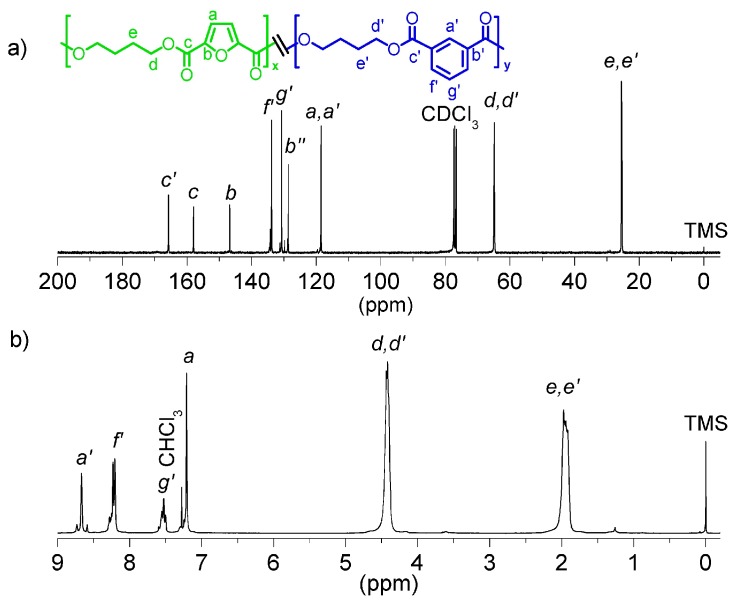
(**a**) ^13^C- and (**b**) ^1^H-NMR of *co*PBF_50_I_50_ with peak assignments.

The polyesters were obtained in good yields (85–93%). The chemical structure and composition of *co*PBF_x_I_y_ copolymers were determined by NMR. Figure 3 shows both ^1^H- and ^13^C-NMR spectra of *co*PBF_50_I_50_ with peak assignments as a representative of the series. NMR spectra for all series are depicted in Figure S5 of SI document. Signals due to the furanic proton *a* and isophthalic protons *f’* were chosen for the determination of the copolyester composition. In general, a good correlation between the feed and the final copolyester composition determined by ^1^H-NMR was found, with slight fluctuations probably due to uncontrolled cycles volatilization (Table 2).

**Figure 4:**
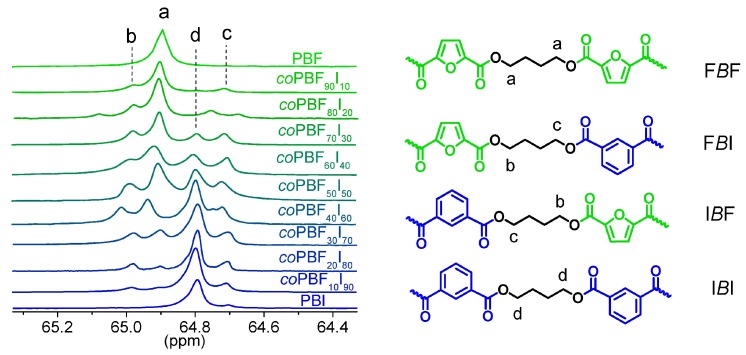
^13^C-NMR spectra of *co*PBF_x_I_y_ copolyesters in the region of the first methylene of the oxybutylene segment.

In contrast, ^13^C-NMR spectra were used for the determination of the copolymer microstructure. Each carbon signal of the butylene segment split into four peaks due to its sensitivity to sequence distribution at the level of dyads (Figure 4). The assignment of the peaks contained in the different dyads was straightforward by comparison to those appearing in both PBF and PBI homopolyesters. By deconvolution of these signals, the dyad content (F*B*F, F*B*I + I*B*F, I*B*I) could be obtained and the number average sequence length and degree of randomness (*R*) could be determined for each copolymer by applying the following expressions [24]:

$${\overset{\text{¯}}{n}}_{\mathrm{BF}}=\frac{FBF+\frac{1}{2}\left(FBI+IBF\right)}{\frac{1}{2}\left(FBI+IBF\right)};{\;\overset{\text{¯}}{n}}_{\mathrm{BI}}=\frac{IBI+\frac{1}{2}\left(FBI+IBF\right)}{\frac{1}{2}\left(FBI+IBF\right)};R=\;\frac{1}{{\overset{\text{¯}}{n}}_{\mathrm{BF}}}+\frac{1}{{\overset{\text{¯}}{n}}_{\mathrm{BI}}}$$

The degree of randomness was near to one with lower values for copolymers with contents in isophthalate units of between 50 mol % and 80 mol %. These values are higher than those that should be expected when only the ROP reaction takes place. In such cases, blocky copolymers and lower values of *R* should be obtained [25]. The observed values indicate that extensive transesterifications took place during polymerization, leading to nearly statistical copolymers (Table 2).

**Figure 5:**
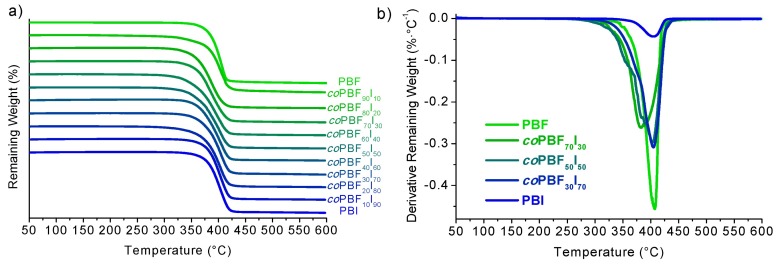
TGA analysis of *co*PBF_x_I_y_. (**a**) Weight loss vs. temperature traces; (**b**) Derivative curves.

The thermal stability of PBF, PBI and their copolyesters was evaluated by TGA under an inert atmosphere. Both, polyesters, and copolyesters were observed to be thermally stable up to 300 °C with onset temperatures above 330 °C, a temperature of maximum decomposition rate close to 400 °C (Figure 5 and Table 3), and remaining weights at 600 °C between 7% and 11%. These values indicate that both homopolyesters and copolyesters have a good thermal stability, and may be processed above their melting temperature without suffering significant thermal degradation.

**Figure 6:**
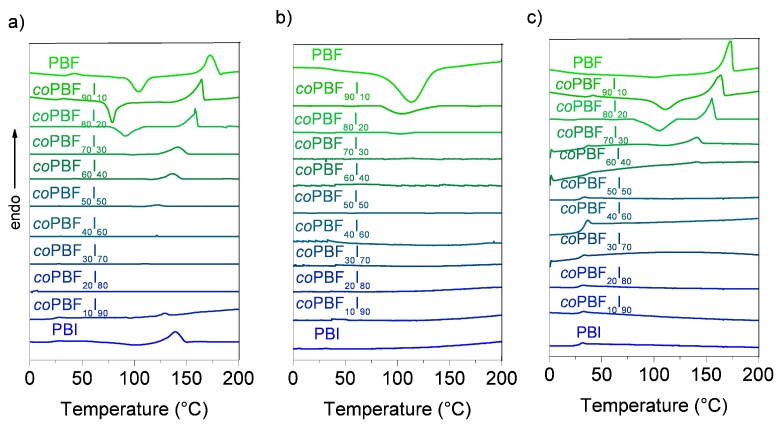
DSC analysis of *co*PBF_x_I_y_ copolyesters. (**a**) First heating; (**b**) cooling from the melt; and (**c**) second heating.

Thermal properties of the copolyesters, such as melting and glass transition temperatures, have been evaluated by DSC. The DSC traces obtained at heating from samples coming directly from synthesis are depicted in Figure 6a–c and data taken from these thermograms are collected in Table 3.

DSC traces recorded at heating from *co*PBF_x_I_y_ samples quenched from the melt showed a single *T*_g_ intermediate between the two homopolyesters, with a value that decreased continuously from 41 to 21 °C with the content of isophthalate units in the copolyester. This feature is usually taken as an indication of the presence of random copolymers or miscible polymers blends. On the other hand, the replacement of furanoate by terephthalate units showed the opposite effect in the copolyesters, with a small increase of *T*_g_ with the content of terephthalate units [21].

The DSC analysis showed that, according to expectations, both PBF and PBI are semicrystalline polyesters with melting temperatures of 173 and 141 °C, and melting enthalpies of 45 and 31 kJ·mol^−1^, respectively. The insertion of butylene isophthalate units in PBF restricted its crystallinity, reducing gradually both the melting temperature and enthalpy as their content increased. In fact, copolyesters with contents between 60 mol % and 80 mol % of isophthalate units showed very low melting enthalpy values at the first heating (≤3 kJ·mol^−1^), and were unable to crystallize upon cooling from the melt. This last effect was not observed for PBF copolyester series containing terephthalate units in the copolyester, and all copolyesters were observed to be semicrystalline [21].

**Figure 7:**
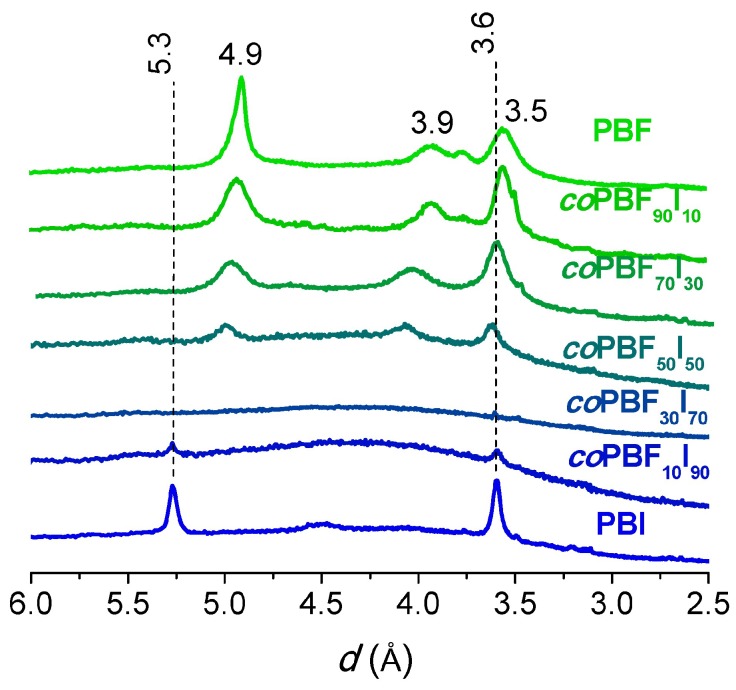
WAXS diffractograms of *co*PBF_x_I_y_.

The intensity profiles of the X-rays scattered by powder pristine samples of representative *co*PBF_x_I_y_ copolyesters and the homopolyesters examined in the present work are compared in Figure 7. In agreement with DSC results, discrete scattering was observed for copolyesters containing up to 50 mol % of isophthalate units, with spacings at around 4.9, 3.9 and 3.5 Å. Peaks broadening with the content of isophthalate units in the copolyester agree with should be expected for the impoverishment of the polymer crystallites, and their slight displacement upwards is probably due to the crystal lattice strain caused by the isophthalate units placed at the crystalline-amorphous interphase. From a comparison of these profiles with those produced by PBF, it can be inferred that semicrystalline copolyesters share the triclinic structure of the homopolyester, a fact that implies the exclusion of the isophthalate units from the crystal lattice [9]. Over 50 mol % of isophthalate units, the copolyesters produced amorphous scattering up to 90 mol %, where the profiles showed weak reflections at 5.3 and 3.6 Å, characteristic of the crystal structure of PBI [26].

**Figure 8:**
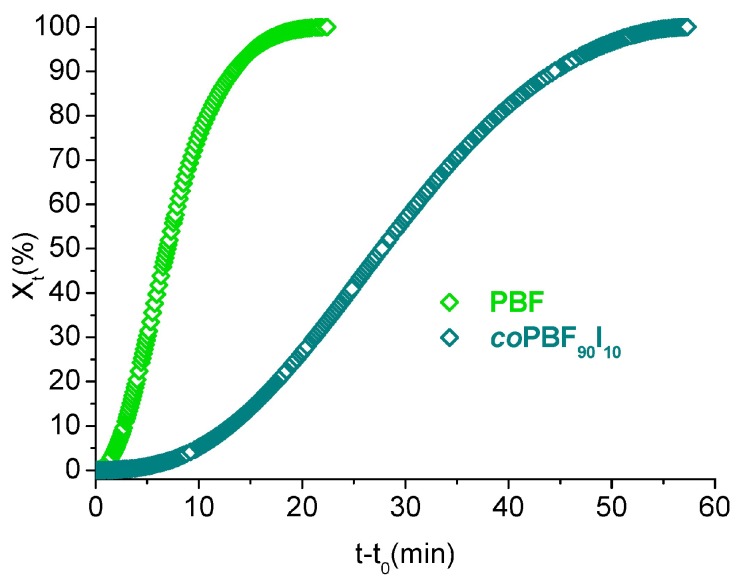
Evolution of the relative crystallinity as a function of time in the isothermal crystallization of PBF and *co*PBF_90_I_10_ at 146 °C.

In order to better understand the effect of the copolymerization on the crystallization behavior, a preliminary isothermal crystallization study has been carried out on PBF homopolymer and a copolymer containing 10 mol % of isophthalate units. Samples that were melted and quenched to 146 °C were isothermally crystallized for one hour at this temperature, and the crystallization enthalpy values generated over time were registered by DSC. It was found that the relative crystallinity (*X*_t_) increased following a sigmoidal trend in both cases (Figure 8), but that the crystallization rate decreased substantially in the *co*PBF_90_I_10_ copolyester.

The crystallization kinetics was analyzed by means of the Avrami approach. Taking the logarithm of both sides of the Avrami equation gives following equation:

$$\log\left(-\ln\left(1-X_{t}\right)\right)=\log\left(K\right)+n\;\log(t-t_{o})$$

where *X_t_* is the fraction of crystallized material, *K* is the temperature-dependent rate constant, *t* and *t_o_* are the elapsed and the onset times respectively, and *n* is the Avrami exponent, indicative of the type of nucleation and dimensionality of crystal growth. Both, *n* and log(*K)* were determined from the slope and the intercept of the linear plot of log(−ln(1 − *X_t_*)) against log(*t − t_o_*), respectively, and the resulting values are compared in Table 3 (Figure S6). These results led us to conclude that a similar nucleation/growing mechanism was operating in the crystallization of the two samples, since close values were obtained for *n* in both cases. In contrast, the crystallization half time increased by a factor of around four in the copolyester, revealing that crystallizability of PBF becomes severely hindered by copolymerization, even for small comonomer contents.

## 4. Conclusions

Partially renewable random poly(butylene furoate) copolyesters (PBF-PBI) containing butylene isophthalate units have been synthesized by ROP of cyclic oligoesters. They were obtained with high molecular weights in good yields, and apparently free from impurities. These results are comparable to those obtained in the preparation of PBF-PBT copolyesters, and proove the suitability of the ROP technique as a general tool to synthesize PBF copolyesters. The crystallinity of the PBF-PBI copolyesters was drastically repressed by the insertion of the isophthalate units. This effect was found to be more efficient than that observed for PBF-PBT copolyesters. The crystallizability, even for small contents of isophthalate units, was also drastically reduced, which opens the possibility to use them, provided that they display good mechanical properties, for applications where a more precise control of the crystallization rate is required.

## Figures and Tables

**Table 1:** Cyclization reaction results.

	Yield (%)	Composition^1^ (2/3/4)	*T*_m_^2^ (°C)	^o^*T*_5%_^2^ (°C)	*T*_d_^2^ (°C)
*c*(BF)_n_	67	61/31/8	147	276	387
*c*(BI)_n_	70	75/15/10	149	330	399

^1^ Relative content (*w*/*w*) of the reaction product in cyclic dimer, trimer and tetramer as measured by HPLC. ^2^ Melting and decomposition temperatures measured by DSC and TGA.

**Table 2:** Results of molecular weight and microstructure analysis for *co*PBF_x_I_y_ copolyesters obtained via ROP.

Copolyester	Yield (%)	x_BF_/y_BI_^1^ (mol/mol)	Molecular Weight ^2^		Dyad Content (mol %) ^3^			Sequence Length ^4^		*R*^4^
			*M_w_*	*Đ*	F*B*F	F*B*I + I*B*F	I*B*I	*n*_BF_	*n*_BI_	
PBF	90	100/0	66,200	1.65	-	-	-	-	-	-
*co*PBF_90_I_10_	88	89/11	66,000	1.50	79.7	18.4	1.7	11.30	1.18	0.94
*co*PBF_80_I_20_	85	81/19	65,800	1.45	72.7	14.1	13.2	9.57	1.50	0.94
*co*PBF_70_I_30_	86	70/30	65,800	1.28	43.1	41.7	14.8	3.06	1.71	0.91
*co*PBF_60_I_40_	85	64/36	65,800	1.30	43.3	42.9	14.1	3.02	1.65	0.93
*co*PBF_50_I_50_	86	48/52	65,200	1.45	25.8	36.8	37.5	2.41	3.04	0.80
*co*PBF_40_I_60_	89	40/60	64,100	1.62	22.1	32.5	45.4	2.36	3.79	0.72
*co*PBF_30_I_70_	91	31/69	63,800	1.78	17.9	27.5	55.4	2.24	5.04	0.75
*co*PBF_20_I_80_	88	18/82	63,200	1.60	5.6	24.2	70.2	1.46	6.79	0.83
*co*PBF_10_I_90_	87	10/90	62,000	1.45	1.7	16.2	82.2	1.21	11.17	0.91
PBI	93	0/100	61,000	1.50	-	-	-	-	-	-

^1^ Determined by ^1^H-NMR. ^2^ Weight-average molar masses in g·mol^−1^ and dispersities determined by GPC. ^3^ Determined by deconvolution of the ^13^C-NMR peaks appearing in the 64.6–65.2 ppm region. ^4^ Number average sequence lengths and degree of randomness (*R*), calculated using the expressions mentioned in the text.

**Table 3:** Thermal properties of *co*PBF_x_I_y_ copolyesters prepared via ROP.

Copolyester	TGA			DSC					Crystallization Kinetics		
					First Heating		Second Heating				
	^o^*T*_d_^1^ (°C)	^ma*x*^*T*_d_ (°C)	*R*_w_ (%)	*T*_g_ (°C)	*T*_m_ (°C)	∆*H (*J·mol^−1^)	*T*_m_ (°C)	∆*H* (J·mol^−1^)	*n*^2^	*lnK*^2^	*t*_1/2_ (min)
PBF	364	407	7	41	173	45	173	39	2.2	−4.4	6.9
*co*PBF_90_I_10_	340	404	11	35	164	41	163	35	2.5	−8.6	27.8
*co*PBF_80_I_20_	338	390	9	30	158	29	156	26	-	-	-
*co*PBF_70_I_30_	342	396	7	28	143	21	142	15	-	-	-
*co*PBF_60_I_40_	330	396	7	28	137	12	137	4	-	-	-
*co*PBF_50_I_50_	334	403	7	27	126	10	-	-	-	-	-
*co*PBF_40_I_60_	355	404	8	27	122	3	-	-	-	-	-
*co*PBF_30_I_70_	352	405	7	27	118	1	-	-	-	-	-
*co*PBF_20_I_80_	336	403	8	26	106	2	-	-	-	-	-
*co*PBF_10_I_90_	363	403	8	24	130	5	129	1	-	-	-
PBI	364	404	8	21	142	31	141	1	-	-	-

^1 o^*T*_d_ obtained at 5% of weight lost. ^2^ Avrami parameters obtained from isothermal crystallization at 146 °C.

## Supplementary Materials

The following are available online at http://www.mdpi.com/2073-4360/10/5/483/s1, Figure S1: (a) ^13^C-NMR, (b) ^1^H-NMR spectra of isophthaloyl chloride, Figure S2: (a) ^1^H-NMR, (b) HPLC and (c) MALDI-ToF of *c*(BF)_n_, Figure S3: (a) ^1^H-NMR, (b) HPLC and (c) MALDI-ToF of *c*(BI)_n_, Figure S4: (a) DSC and (b) TGA analysis of *c*(BF)_n_ and *c*(BI)_n_, Figure S5: (a) ^13^C- and (b) ^1^H-NMR of *co*PBF_x_I_y_, Figure S6: Double logarithmic plot of the Avrami equation for experimental data recorded from the isothermal crystallization of *co*PBF_90_I_10_ and PBF.

## References

[1] Miller S.A. (2014). Sustainable polymers: Replacing polymers derived from fossil fuels. Polym. Chem..

[2] Gandini A. (2011). The irruption of polymers from renewable resources on the scene of macromolecular science and technology. Green Chem..

[3] Gandini A., Lacerda T.M. (2015). From monomers to polymers from renewable resources: Recent advances. Prog. Polym. Sci..

[4] Corma A., Iborra S., Velty A. (2007). Chemical routes for the transformation of biomass into chemicals. Chem. Rev..

[5] Coates G.W., Hillmyer M.A. (2009). A virtual issue of macromolecules: Polymers from renewable resources. Macromolecules.

[6] Sousa A.F., Vilela C., Fonseca A.C., Matos M., Freire C.S.R., Gruter G.J.M., Coelhob J.F.J., Silvestre A.J.D. (2015). Biobased polyesters and other polymers from 2,5-furandicarboxylic acid: A tribute to furan excellency. Polym. Chem..

[7] Gandini A., Lacerda T.M., Carvalho A.J.F., Trovatti E. (2016). Progress of polymers from renewable resources: Furans, vegetable oils, and polysaccharides. Chem. Rev..

[8] Papageorgiou G.Z., Papageorgiou D.G., Terzopoulou Z., Bikiaris D.N. (2016). Production of bio-based 2,5-furan dicarboxylate polyesters: Recent progress and critical aspects in their synthesis and thermal properties. Eur. Polym. J..

[9] Zhu J.H., Cai J.L., Xie W.C., Chen P.H., Gazzano M., Scandola M., Gross R.A. (2013). Poly(butylene 2,5-furan dicarboxylate), a biobased alternative to PBT: Synthesis, physical properties, and crystal structure. Macromolecules.

[10] Kint D.P.R., Muñoz-Guerra S. (2003). Modification of the thermal properties and crystallization behaviour of poly(ethylene terephthalate) by copolymerization. Polym. Int..

[11] Zheng M.Y., Zang X.L., Wang G.X., Wang P.L., Lu B., Ji J.H. (2017). Poly(butylene 2,5-furandicarboxylate-epsilon-caprolactone): A new bio-based elastomer with high strength and biodegradability. Express Polym. Lett..

[12] Wu B.S., Xu Y.T., Bu Z.Y., Wu L.B., Li B.G., Dubois P. (2014). Biobased poly(butylene 2,5-furandicarboxylate) and poly(butylene adipate-co-butylene 2,5-furandicarboxylate)s: From synthesis using highly purified 2,5-furandicarboxylic acid to thermo-mechanical properties. Polymer.

[13] Oishi A., Iida H., Taguchi Y. (2010). Synthesis of poly(butylene succinate) copolymer including 2,5-furandicarboxylate. Kobunshi Ronbunshu.

[14] Papageorgiou G.Z., Papageorgiou D.G. (2017). Solid-state structure and thermal characteristics of a sustainable biobased copolymer: Poly(butylene succinate-co-furanoate). Thermochim. Acta.

[15] Morales-Huerta J.C., Ciulik C.B., Martínez de Ilarduya A., Muñoz-Guerra S. (2017). Fully bio-based aromatic-aliphatic copolyesters: Poly(butylene furandicarboxylate-co-succinate)s obtained by ring opening polymerization. Polym. Chem..

[16] Morales-Huerta J.C., Martínez de Ilarduya A., Muñoz-Guerra S. (2018). Blocky poly(ε-caprolactone-co-butylene 2,5-furandicarboxylate) copolyesters via enzymatic ring opening polymerization. J. Polym. Sci. Pol. Chem..

[17] Brunelle D.J. (2004). Synthesis and polymerization of cyclic polyester oligomers. Modern Polyesters: Chemistry and Technology of Polyesters and Copolyesters.

[18] Hodge P. (2014). Entropically driven ring-opening polymerization of strainless organic macrocycles. Chem. Rev..

[19] Strandman S., Gautrot J.E., Zhu X.X. (2011). Recent advances in entropy-driven ring-opening polymerizations. Polym. Chem..

[20] Morales-Huerta J.C., Martínez de Ilarduya A., Muñoz-Guerra S. (2017). A green strategy for the synthesis of poly(ethylene succinate) and its copolyesters via enzymatic ring opening polymerization. Eur. Polym. J..

[21] Morales-Huerta J.C., Martínez de Ilarduya A., Muñoz-Guerra S. (2016). Sustainable aromatic copolyesters via ring opening polymerization: Poly(butylene 2,5-furandicarboxylate-co-terephthalate)s. ACS Sustain. Chem. Eng..

[22] Brunelle D.J., Bradt J.E., Serth-Guzzo J., Takekoshi T., Evans T.L., Pearce E.J., Wilson P.R. (1998). Semicrystalline polymers via ring-opening polymerization: Preparation and polymerization of alkylene phthalate cyclic oligomers. Macromolecules.

[23] Morales-Huerta J.C., Martínez de Ilarduya A., Muñoz-Guerra S. (2016). Poly(alkylene 2,5-furandicarboxylate)s (PEF and PBF) by ring opening polymerization. Polymer.

[24] Randall J. (2012). Polymer Sequence Determination: Carbon-13 NMR Method.

[25] Kamau S.D., Hodge P., Williams R.T., Stagnaro P., Conzatti L. (2008). High throughput synthesis of polyesters using entropically driven ring-opening polymerizations. J. Comb. Chem..

[26] Sanz A., Nogales A., Ezquerra T.A., Lotti N., Munari A., Funari S.S. (2006). Order and segmental mobility during polymer crystallization: Poly(butylene isophthalate). Polymer.

