# Supplementary material for: Partially Renewable Poly(butylene 2,5-furandicarboxylate-co-isophthalate) Copolyesters Obtained by ROP
Source: Polymers (Basel). 2018 Apr 28;10(5):483. doi: 10.3390/polym10050483 (PMC6415514; doi:10.3390/polym10050483)
Supplement: Supplementary file 1 [file polymers-10-00483-s001.pdf]

Partially renewable poly(butylene 2,5-furandicarboxylate-*co*-isophthalate) copolyesters obtained by entropically-driven ROP

**Juan Carlos Morales-Huerta, Antxon Martínez de Ilarduya*
and Sebastián Muñoz-Guerra***

Department d'Enginyeria Química, Universitat Politècnica de Catalunya, ETSEIB, Diagonal 647,8028 Barcelona, Spain

Correspondence to: A, Martínez de Ilarduya (E-mail: antxon.martinez.de.ilarduia@upc.edu)
S. Muñoz-Guerra (E-mail: sebastian.munoz@upc.edu)

Contents:

Figure S1. a) ^{13}C NMR, b) ^{1}H NMR spectra of isophthaloyl chloride.

Figure S2. a) ^{1}H NMR, b) HPLC and c) MALDI-ToF of *c*$(BF)_n$.

Figure S3. a) ^{1}H NMR, b) HPLC and c) MALDI-ToF of *c*$(BI)_n$.

Figure S4. a) DSC and b) TGA analysis of *c*$(BF)_n$ and *c*$(BI)_n$.

FigureS5. a) ^{13}C and b) ^{1}H NMR of *co*PBF_xI_y.

FigureS6. Double logarithmic plot of crystallization of $coPBF_{90}I_{10}$ compared with PBF.

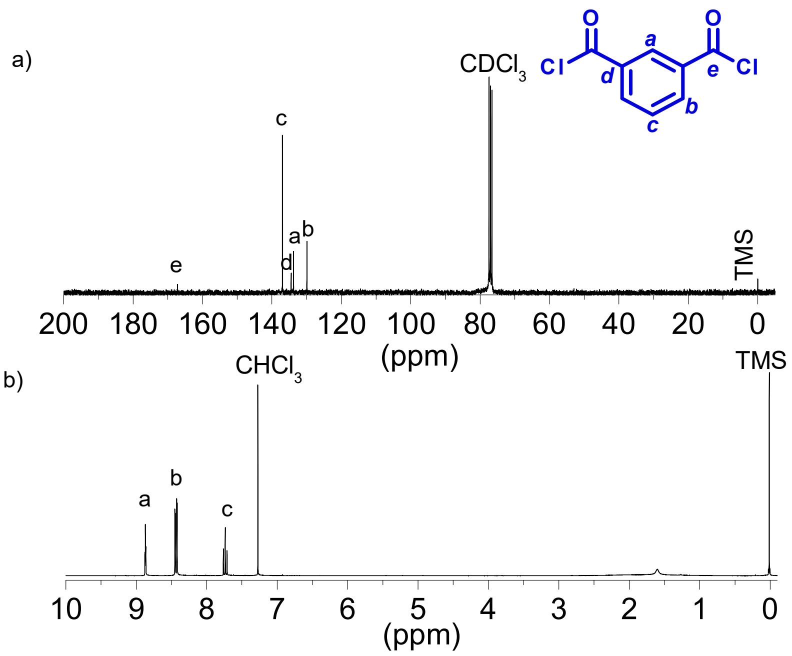

Figure S1. a) ^{13}C NMR, b) ^{1}H NMR spectra of isophthaloyl chloride.

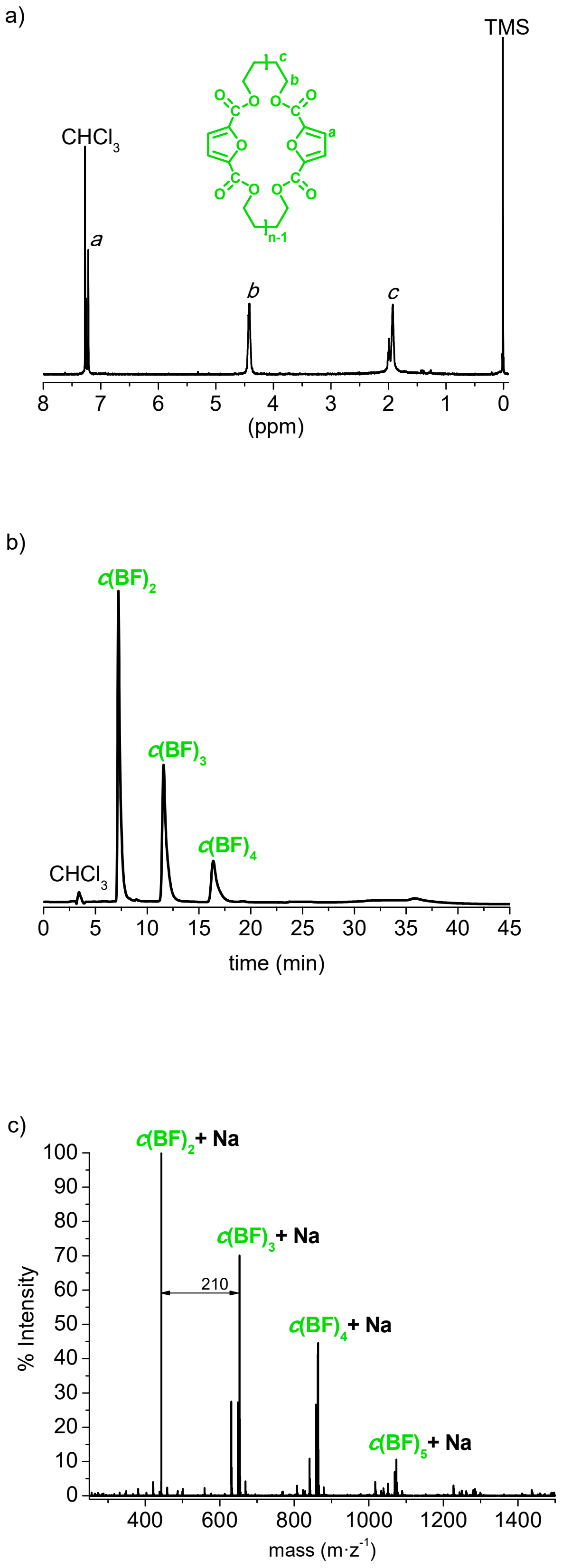

Figure S2. a) ^{1}H NMR, b) HPLC and c) MALDI-ToF of c(BF)$_n$.

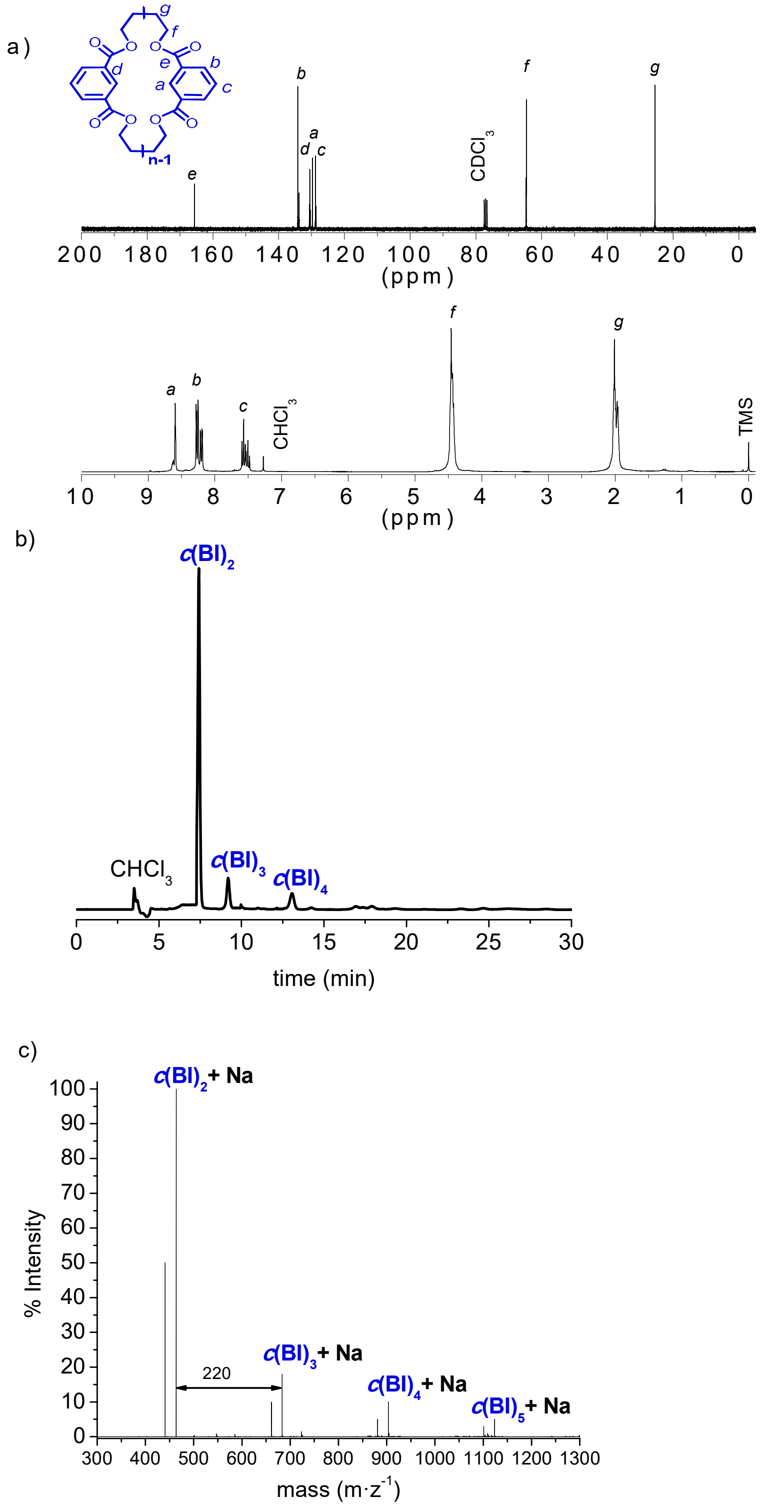

Figure S3. a) ^{1}H NMR, b) HPLC and c) MALDI-ToF of $c(\mathrm{BI})_n$.

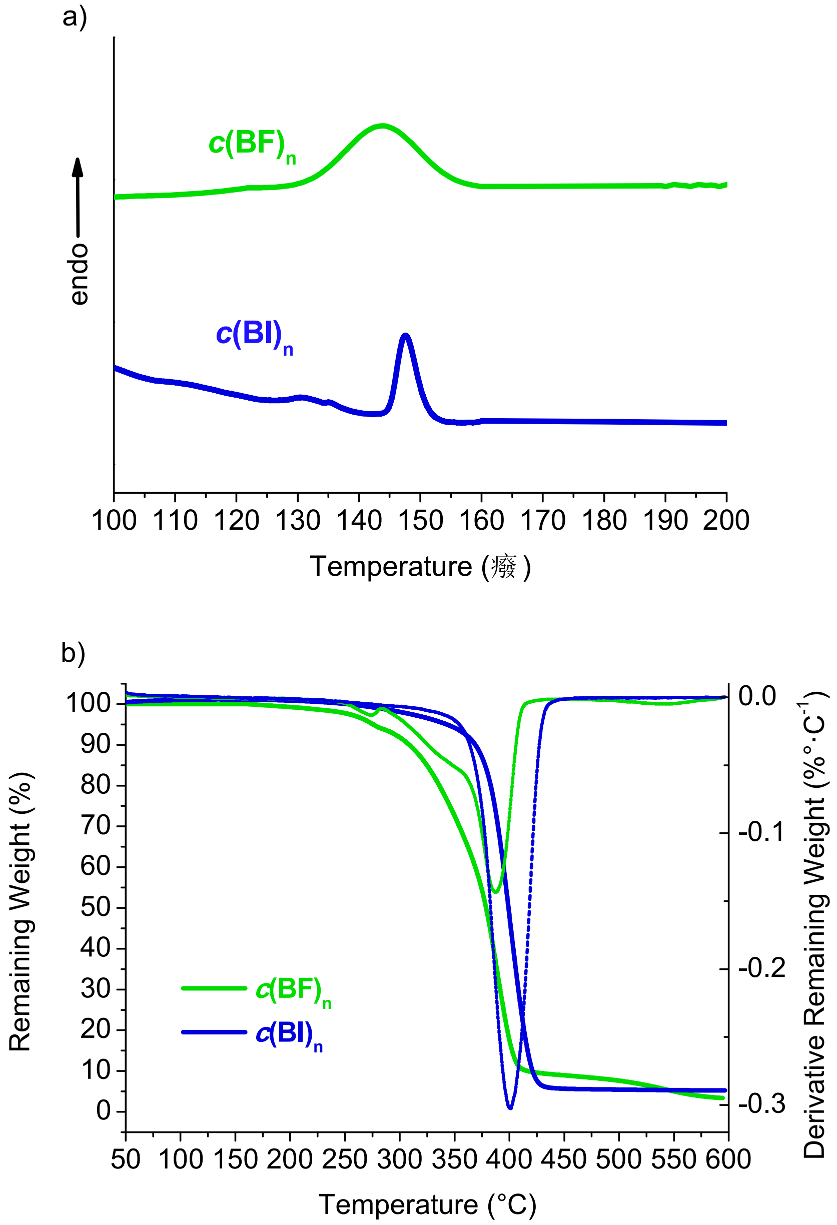

Figure S4. a) DSC and b) TGA analysis of *c*(BF)$_n$ and *c*(BI)$_n$.

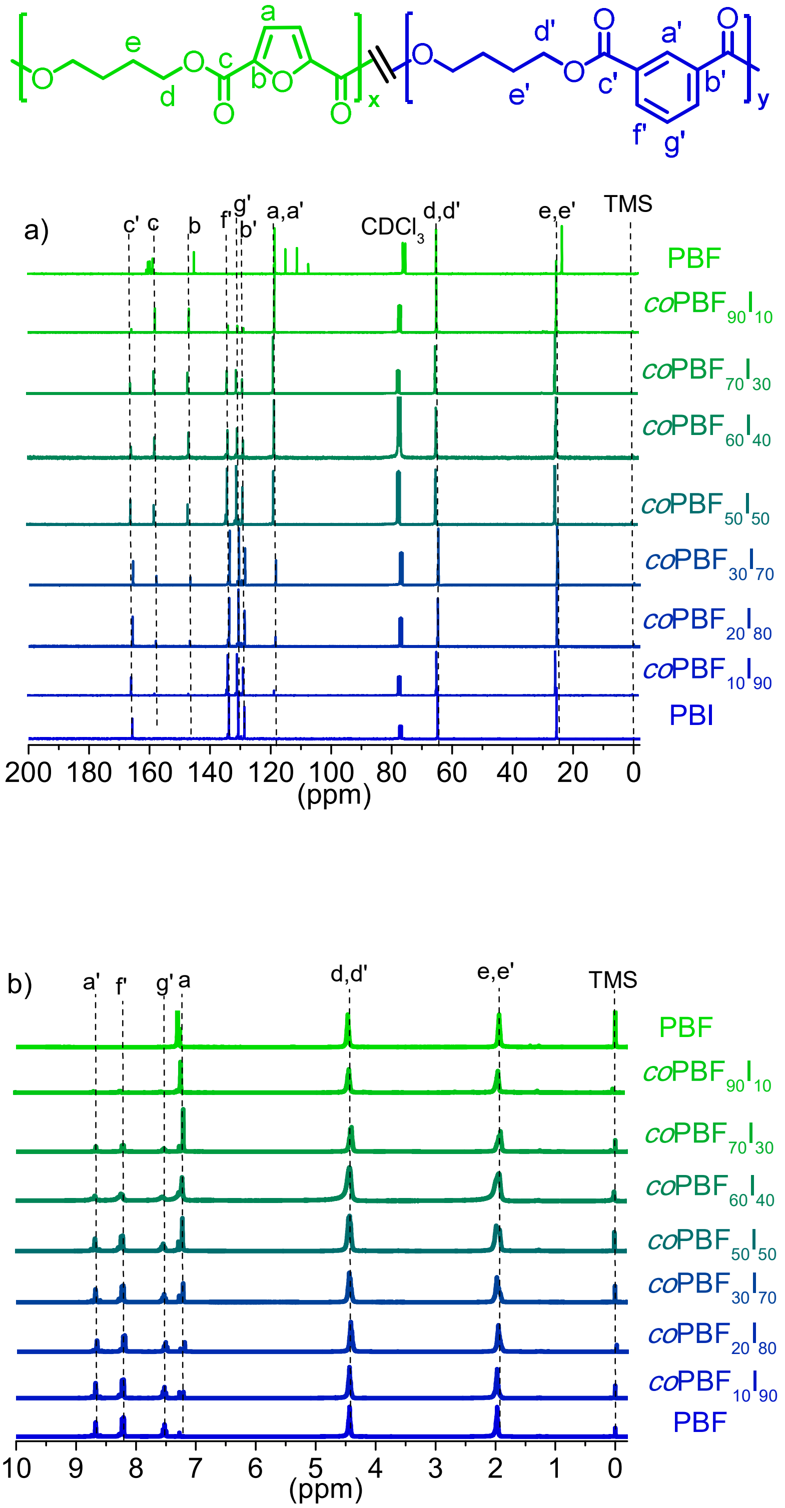

Figure S5. a) ^{13}C and b) ^{1}H NMR of *co*PBF$_x$I$_y$.

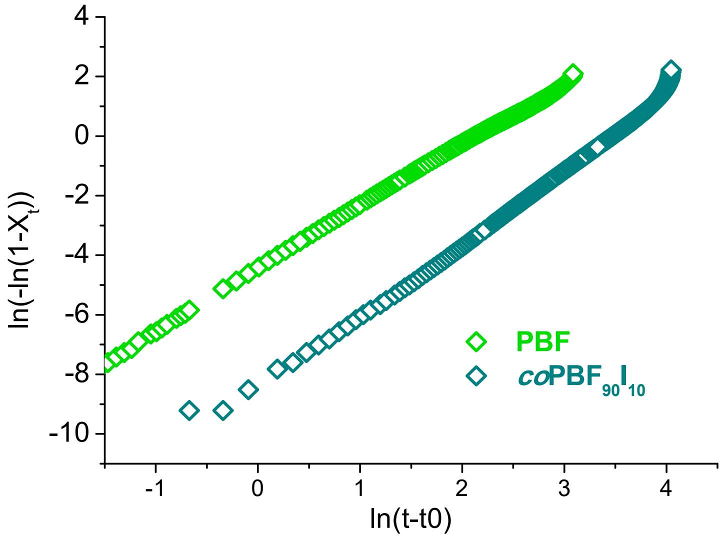

Figure S6. Double logarithmic plot of the Avrami equation for experimental data recorded from the isothermal crystallization of *co*PBF$_{90}$I$_{10}$ and PBF.
